# Wearable Cardioverter-defibrillators for the Prevention of Sudden Cardiac Death: A Meta-analysis

**DOI:** 10.19102/icrm.2018.090506

**Published:** 2018-05-15

**Authors:** Elaine Nguyen, Erin R. Weeda, Christine G. Kohn, Benjamin A. D’Souza, Andrea M. Russo, Stacey Noreika, Craig I. Coleman

**Affiliations:** ^1^Department of Pharmacy Practice, Idaho State University College of Pharmacy, Meridian, ID, USA; ^2^Department of Pharmacy Practice, The Medical University of South Carolina, Charleston, SC, USA; ^3^Department of Pharmacy Practice, University of Connecticut, Storrs, CT, USA; ^4^Hospital of the University of Pennsylvania, Philadelphia, PA, USA; ^5^Cooper Medical School of Rowan University, Camden, NJ, USA

**Keywords:** LifeVest, sudden cardiac death, ventricular fibrillation, ventricular tachycardia, wearable cardioverter-defibrillator

## Abstract

Wearable cardioverter-defibrillators (WCDs) protect patients from sudden cardiac death (SCD) by detecting and treating life-threatening ventricular tachycardia/fibrillation (VT/VF). Recently, two large studies evaluating WCDs were published. However, the results of older and newer studies have yet to be systematically summarized. The objective of the current study was to conduct a meta-analysis assessing the use and effectiveness of WCDs. We searched MEDLINE and Scopus (January 1998–July 2017) as well as the gray literature. We included registry/observational studies that (1) evaluated adult patients using WCDs; (2) provided data on one or more outcomes of interest; and (3) were full-text studies published in English. We calculated pooled incidence and/or rate [with 95% confidence intervals (CIs)] estimates from nonoverlapping populations using a random-effects meta-analysis model. Statistical heterogeneity was assessed via the I^2^ statistic. We identified 11 studies (19,882 patients) with nonoverlapping populations/endpoints; seven of them evaluated WCD use across various indications, while the remaining studies restricted their focus to a single indication. Most of the studies were retrospective (82%) and multicenter (64%) in nature, with 45% using manufacturers’ registry data. The median duration of WCD use was three or more months in nine (82%) studies, and daily wear time ranged from a mean/median of 17 hours to 24 hours per day across included studies. Seven (64%) studies reported a mean/median daily wear time of more than 20 hours. This meta-analysis showed that the incidences of all-cause and SCD-related mortality among WCD patients were 1.4% (95% CI: 0.7%–2.4%) and 0.2% (95% CI: 0.1%–0.3%), respectively. VT/VF occurred in 2.6% (95% CI: 1.8%–3.5%) of patients. Across patients, 1.7% (95% CI: 1.4%–2.0%) received appropriate WCD treatment, corresponding to a rate of 9.1 patients/100 person-years (95% CI: 6.2–11.9 patients/100 person-years). Successful VT/VF termination following appropriate treatment occurred in 95.5% of patients (95% CI: 92.0%–98.0%) and the incidence of inappropriate treatment was infrequent (0.9%; 95% CI: 0.5%–1.4%). A moderate-to-high degree of statistical heterogeneity was observed in pooled analyses of mortality, VT/VF occurrence, and appropriate/inappropriate treatment (I^2^ ≥ 41% for all). In conclusion, WCDs appear to be successful in terms of terminating VT/VF in patients with an elevated risk of SCD and are appropriate for use while long-term risk management strategies are being identified.

## Introduction

Sudden cardiac death (SCD) accounts for more than 300,000 deaths in the United States each year.^1^ Ventricular tachyarrhythmias [ventricular tachycardia (VT) or ventricular fibrillation (VF)] are the first recorded rhythms in more than 20% of all out-of-hospital cardiac arrests.^2^ Time to defibrillation is a critical factor in the reduction of SCD attributable to VT/VF, with the probability of survival decreasing by 3% to 10% for each minute defibrillation is delayed.^3^

Wearable cardioverter-defibrillators (WCDs) detect and deliver timely defibrillation to patients experiencing sustained VT/VF to prevent SCD. According to a 2016 science advisory prepared by the American Heart Association (AHA), WCDs can be used to provide automated defibrillation therapy in high-risk patients who are not candidates for an implantable cardioverter-defibrillator (ICD), including those at elevated risk of ventricular arrhythmias for a transient period who may experience clinical improvement and those with an indication for an ICD but a contraindication to immediate device implantation.^4^

The effectiveness of WCDs has been documented in numerous studies since 1998, including in two large, population-based registry studies published after the 2016 AHA science advisory on WCD use was released.^4–7^ To date, however, the results of these studies have not been systematically summarized. Therefore, we performed a meta-analysis of studies assessing the occurrence of sustained ventricular tachyarrhythmic events in WCD users and evaluating the use and effectiveness of WCDs among at-risk cardiac patients.

## Methods

This report was written to conform to the reporting standards described in the Preferred Reporting Items for Systematic Reviews and Meta-Analyses statement and the Meta-Analysis of Observational Studies in Epidemiology proposal.^8,9^

For this meta-analysis, we identified research studies that (1) evaluated adult patients using WCDs; (2) provided data on one or more outcomes of interest (eg, all-cause and VT/VF-related mortality, VT/VF occurrence, appropriate and inappropriate shock therapy, and successful termination of VT/VF); and (3) were full-text studies published in the English language. Eligible studies evaluating patients who received a WCD for a single indication or evaluating the use and effectiveness of WCDs in patients across a number of indications (ie, mixed indication studies) were included. Studies meeting these criteria were real-world registry or observational studies. As we anticipated a portion of the identified studies would be using the same manufacturer registry (US/Europe) as a source of data, care was taken to prevent inclusion/statistical pooling of studies with overlapping patient populations in order to avoid biased conclusions due to correlations in study results. If studies were identified as having overlapping populations (based upon month/year and indication for WCD use), the study with the greatest generalizability (ie, the one with the broadest set of allowable indications, largest sample size, and/or a multicenter nature) was included. Importantly, we defined that studies that could initially have been excluded due to overlapping populations could still be included in this meta-analysis if they reported data on an original endpoint not found in their counterpart study.

We performed literature searches of both the MEDLINE and Scopus bibliographic databases from January 1998 through July 2017 to identify relevant studies. Our MEDLINE search strategy is available in **Table 1**. Database searches were augmented with a manual search of the citations listed on the manufacturer’s website^10^ and a Google search (from which we reviewed the first 200 citations). Finally, backward citation tracking of references from identified studies and review articles was performed to identify additional relevant studies. At both the citation title/abstract and full-text article review stages, two independent study investigators reviewed each potential report for inclusion/exclusion, with disagreements resolved by discussion or with input from a third investigator.

For all studies deemed eligible for inclusion into this meta-analysis, relevant study demographic and validity characteristics (author and year of publication, sample size, study design, country and number of centers, data source and sampling dates, indication for WCD use, mean/median patient age, patient sex, baseline ejection fraction, duration of WCD use, daily wear time, and end of use) and endpoint data were extracted by two investigators using a standardized worksheet, with disagreement resolved via discussion or with input from a third investigator.

Key characteristics of each included study were descriptively summarized in tables. In order to assess the precision of binomial endpoint data, exact Clopper-Pearson 95% confidence intervals (CIs) were estimated for incidences, while Poisson 95% CIs were estimated for rates. We pooled incidence and/or rates (episodes per 100 person-years of follow-up) with accompanying 95% CIs for each a priori endpoint using a traditional random-effects meta-analysis model. The presence of statistical heterogeneity was assessed via the I^2^ statistic, with I^2^ values of < 25%, 25% to 75%, and > 75% indicative of small, moderate, and high degrees of between-study heterogeneity, respectively. The likelihood of publication bias was evaluated using Egger’s weighted regression statistic p-value. All statistical analyses were performed using StatsDirect version 2.7.6 (StatsDirect Ltd., Cheshire, England).

## Results

**Figure 1:**
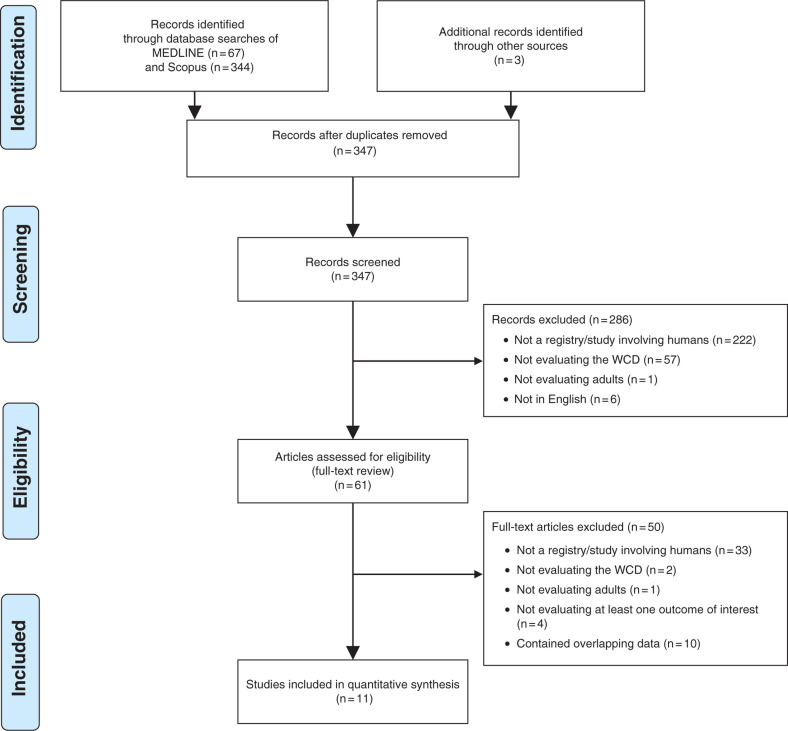
Flow diagram of record inclusion/exclusion criteria.

Our systematic literature search yielded 411 citations, with three citations identified through additional sources **(Figure 1)**. After title/abstract screening and full-text assessment, 11 studies with nonoverlapping populations and/or endpoints evaluating a cumulative total of 19,882 patients (range: 8–8,453 patients) were included **(Table 2)**.^5,6,11–19^ Most studies were retrospective (n = 9; 82%) and multicenter (n = 7; 64%) in nature, with five studies (45%) incorporating data from the manufacturer’s registry. Of the studies considered, 56% evaluated patients treated in the US (n = 6), while 36% evaluated patients treated in Germany (n = 4). Seven studies (64%) were mixed indication studies evaluating the following populations: early post-myocardial infarction (MI)/revascularization (range: 11%–64%); heart failure/cardiomyopathy (range: 34%–86%); explanted ICD (range: 10%–38%); history of VT/VF (range: 19%–89%); myocarditis (range: 10%–13%); genetic conditions (range: 1%–13%); heart transplant candidates (range: 0.7%–6%); and miscellaneous conditions (range: 2%–13%). Of the remaining studies, two (18%) were restricted to focusing on early post-MI/revascularization, one (9%) to heart failure/cardiomyopathy, and one (9%) to genetic conditions, respectively. Median duration of WCD use was three or more months in nine (82%) studies and daily wear time ranged from a mean/median of 17 hours/day to 24 hours/day across included studies. Seven (64%) studies reported a mean/median daily wear time of more than 20 hours. Premature discontinuation of the device was reported in five studies and occurred in ≤ 14% of patients in all but one of the earliest studies by Feldman et al. (24%).^19^

Results of the meta-analysis showed that the incidences of all-cause and VT/VF-related mortality were 1.4% (95% CI: 0.68%–2.4%) and 0.2% (95% CI: 0.1%–0.3%), respectively **(Table 3)**. VT/VF occurred in 2.6% (95% CI: 1.8%–3.5%) of patients. A total of 1.7% (95% CI: 1.39%–2.15%) of patients received appropriate WCD therapy, corresponding to a rate of 9.1 patients/100 person-years (95% CI: 6.2–11.9 patients/100 person-years). Successful VT/VF termination following appropriate shock therapy occurred in 95.5% of patients (95% CI: 92.0%–98.0%), while the incidence of inappropriate shock therapy was less frequent (0.9%; 95% CI: 0.5%–1.4%). A moderate-to-high degree of statistical heterogeneity was observed in pooled analyses of all-cause and VT/VF-related mortality and appropriate and inappropriate shock therapy (I^2^ ≥ 41% for all). A low degree of statistical heterogeneity was observed in our analysis of successful VT/VF termination (I^2^ = 0%). Egger’s p-values suggested a low probability of publication bias in all analyses (p ≥ 0.35 for all endpoint analyses).

## Discussion

In this meta-analysis of nearly 20,000 at-risk cardiac patients managed in real-world settings, VT/VF-related mortality occurred infrequently (0.2%) among WCD users. In approximately a three-month median period of use, 2.6% of patients experienced a VT/VF occurrence, with successful VT/VF termination achieved in 95% of patients who required defibrillation. Inappropriate shocks were rare, occurring in < 1% of patients.

I^2^ values quantify the percentage of variation across studies not due to chance alone.^20^ The moderate-to-high I^2^ values seen suggest that statistical heterogeneity existed in our pooled results for all but the VT/VF termination endpoint. This variation was not surprising given the clinical diversity in patients included in these studies. For example, most studies included patients with mixed indications for WCDs. WCD indication is known to impact outcomes, as seen in an analysis that stratified WCD users by traditional primary indications (eg, ICD explants, history of VT/VF, inherited cardiac disorders) and primary prevention indications (eg, after MI with reduced ejection fraction after revascularization, reduced ejection fraction with nonischemic cardiomyopathy).^17^ The probability of higher three-month and three-year mortality rates was observed in traditional versus primary prevention patients (hazard ratio: 4.32, 95% CI: 2.50–7.49 and hazard ratio: 2.28, 95% CI: 1.82–2.85, respectively). It is important to remember that the I^2^ statistic should be interpreted with caution given its known imprecision.^21^ Due to the moderate-to-high I^2^ values observed for many endpoints, we employed a random-effects model to pool estimates in our meta-analysis.

According to a science advisory prepared by the AHA and endorsed by the Heart Rhythm Society and European Society of Cardiology (ESC), WCD use is reasonable when there is an indication for an ICD but a temporary contraindication/interruption (eg, a waiting period, infection) to ICD care exists (class IIa), or when patients are awaiting more definitive treatment (eg, cardiac transplant candidate) (class IIa).^4,22^ Moreover, this same guidance states that the WCD may also be considered in patients with a transient SCD risk that may decrease with clinical improvement (eg, ischemic cardiomyopathy with recent revascularization, newly diagnosed nonischemic cardiomyopathy in patients initiated on evidence-based treatment, or secondary cardiomyopathy caused by a treatable factor) (class IIb) or in circumstances in which ICDs reduce SCD but not overall survival (eg, ~40 days following an MI) (class IIb).

Such recommendations and published reviews often note that no randomized controlled trials to date have evaluated the use and efficacy of WCDs. Thus, our meta-analysis consisted entirely of prospective and retrospective observational studies. However, studying medical devices in a randomized clinical trial can be problematic. The US Food and Drug Administration in a recent draft guidance document stated that randomized trials of devices present challenges due to ethical issues and the “realities of medical device innovation and development cycles.”^23^ The document goes on to state that real-world device data, when methodologically sound, may offer “similar information with comparable or even superior characteristics” to those of clinical trial data. WCDs are currently being evaluated in the ongoing, randomized Vest Prevention of Early Sudden Death Trial (VEST) (NCT01446965).

Since the AHA science advisory and the ESC guidelines were written, two large, population-based WCD registry studies assessing more than 8,000 patients have been published.^5,6^ Kutyifa et al. used a prospective registry of 2,000 WCD patients in the US, while Wäßnig et al. conducted a retrospective analysis of more than 6,000 patients in Germany. These studies evaluated broad indications, including 3,883 dilated cardiomyopathy/nonischemic cardiomyopathy patients and 2,430 ischemic cardiomyopathy patients. The addition of these studies to the available literature nearly doubled the amount of available, nonoverlapping WCD patient data that were available to the authors of the 2016 science advisory. These data were included in the present analysis.

WCDs are designed to prevent unnecessary SCD from VT/VF. These devices generally have successful resuscitation rates of more than 97%. WCDs were developed as a short-term protection method to be deployed in high-risk patients, including those within the 40-day or 90-day “ICD waiting period.” While WCDs are often compared to ICDs, there are several important distinctions.

First, the alternative approach to the LifeVest (Zoll Medical Corporation, Chelmsford, MA, USA) is bystander cardiopulmonary resuscitation (CPR) [with or without automated external defibrillator (AED) application]. Both of these options depend on the sudden cardiac arrest (SCA) event being witnessed by people able and willing to perform resuscitation. Unfortunately, the majority of out-of-hospital cardiac arrests occur at home, with more than half of cases going unwitnessed, making bystander CPR a poor approach for patients at high risk of SCA.^24^ Therefore, it is not surprising that only one in 10 people survive an SCA event, and the chances of survival with good neurological function are even less.^13^ Moreover, the Home Use of Automatic External Defibrillators to Treat SCA (HAT) study compared having an AED in the home followed by emergency medical services activation to emergency medical services activation alone and found no significant difference in mortality.^25^

The most critical factor to survival following SCA is time to defibrillation. Reliance on bystander CPR is not an issue for in-hospital SCA situations wherein patients are more closely monitored; however, even in this environment, survival from SCA has increased very little from 18.1% in 2000 to 2003 to 21.4% in 2007 to 2010.^26^ A study of the National Registry of Cardiopulmonary Resuscitation examined survival of in-hospital SCA and found that survival rates were higher when defibrillation occurred within three minutes.^27^ Experts estimate that, for every minute that elapses during an SCA event, mortality increases by 10%.^28^ In contrast to these poor survival rates following SCA, short-term survival rates with the LifeVest (Zoll Medical Corporation, Chelmsford, MA, USA) exceed 95%, largely due to the fact that defibrillation occurs within one minute and is not dependent on bystander intervention. Based on the high mortality rate of bystander CPR/defibrillation in comparison with the efficacy of defibrillation using a WCD, it could be considered unethical to pursue multiple randomized clinical trials of the WCD versus no WCD.

Second, the issue of ideal timing for ICD implantation is still unclear. Despite the high risk of SCA in some patients, such as those who are post-MI or who have newly diagnosed heart failure with low ejection fraction, previous studies have found little, if any, benefit with early ICD implantation.^29,30^ Also, guidelines-directed medical therapy has improved over the past decade. With newer agents and better diagnostics, physicians are able to provide better care for these populations. The patient’s condition and the risk of SCA can change, as many patients have shown improvement in ejection fraction during the first two to three months.^31,32^ The ideal duration of guidelines-directed medical therapy prior to prophylactic ICD implantation remains uncertain and recent data demonstrate that a relevant proportion of patients with newly diagnosed heart failure may show recovery of left ventricular function beyond three months after the initiation of heart failure therapy.^33^ A WCD provides protection during this high-risk transition period until the patient recovers or his or her physician determines that a continued long-term risk is present that requires protection with an ICD. The temporary protection provided by a WCD is important because SCD risk is at its highest early after an acute cardiac event when alternative therapies, such as ICDs, have not been demonstrated to be advantageous.^34^ WCDs also provide SCD protection for those patients in whom an ICD must be removed or deactivated (eg, due to lead fracture noise).

While defibrillators can deliver life-saving therapy, one risk of these devices, whether external or implanted, is inappropriate shocks. Our analysis found inappropriate shocks to be rare (having an incidence of 0.3% per month during a follow-up of three months or less), exhibiting high sensitivity (90%–100%) and specificity (98%–99%).^4,5,17^ In comparison, during the first six months of ICD use, the inappropriate shock rate has been shown to range between 0.6% and 1.5% per month^35^—a rate that is at least double that observed with WCD use. This difference may be due to the ability of conscious patients to use the response buttons of the WCD to withhold unnecessary treatment. Another reason for this difference may relate to the longer detection window seen with a WCD versus with an ICD, allowing for better rhythm discrimination or tachycardia termination.

Another concern expressed by physicians less familiar with WCDs is patients’ unwillingness to wear them. Our meta-analysis found high patient compliance, with more than half of the included studies reporting a mean/median daily wear time exceeding 20 hours/day. These good compliance rates, especially those in more recent studies (the median daily wear time was 23.1 hours and 22.5 hours in Wäßnig et al.^5^ and Kutyifa et al.,^6^ respectively), may be a result of the increased comfort seen with newer generations of WCDs because they are smaller and weigh less than the initial WCDs used in Feldman et al.^19^

Our analysis has limitations worth noting. First, we had to exclude several studies with overlapping populations in order to prevent bias caused by correlations in study results. However, we attempted to maximize external validity by selecting those studies with the greatest generalizability. Moreover, several of the selected studies used data from registries maintained by WCD manufacturers. Next, we were unable to perform a meta-analysis on other important outcome measures such as patient quality-of-life, patient satisfaction, and costs, as few studies reported this information. In addition, we chose not to perform a formal assessment of study quality given the characteristics of the included studies. Lastly, we did not include non-English language studies due to the language limitations of the study investigators. As with any meta-analysis, we were unable to rule out the potential for publication bias. This being said, Egger’s weighted regression statistics suggested a lower probability of publication bias.

## Conclusions

VT/VF remains an important and potentially avoidable cause of SCD in high-risk patients. WCDs are successful in terminating VT/VF in patients with an elevated risk of SCD and appear to be appropriate for use while long-term risk management strategies are being determined.

## Figures and Tables

**Table 1:** MEDLINE Literature Search Keywords

1. “wearable cardioverter defibrillator”
2. “wearable card* defibrillator”
3. “wearable external cardiac defibrillator”
4. “wearable and automatic external defibrillator”
5. “LifeVest”
6. “Life Vest”
7. “wearable defibrillator”
8. “external defibrillator jacket”
9. “defibrillator vest”
10. “OR/1–9”

The * is the truncation symbol in MEDLINE, sometimes referred to as a “wildcard.”

**Table 2:** Characteristics of the Included Studies

Study	Number of Participants	Study Design	Center(s) Involved, Country	Data Source (Sample Dates)	Indication(s) for WCD		Age: Mean ± SD (Median)	Male Sex	Baseline EF: Mean ± SD (Median)	WCD Use: Mean ± SD (Median)	Compliance: Mean ± SD (Median, 25%–75% Range)	Premature Discontinuation^*^
Wäßnig et al.^5^	6,043	R	Multicenter, Germany	Zoll database^†^ (April 2010–October 2013)	•	DCM (37%)	(57 years)	79%	NR	(59 days)	(23.1, 21.0–23.7)	NR
					•	ICM with CAD and low EF^‡^ (27%)					
					•	NICM (12%)					
					•	Explanted ICD (12%)					
					•	Myocarditis (10%)					
					•	Genetic disease (1%)					
					•	Heart transplant candidate (0.7%)					
					•	Heart failure (0.5%)					
Kutyifa et al.^6^	2,000	P	Multicenter, US	WEARIT-II Registry (August 2011–February 2014)	•	NICM (46%)	(62 years)	70%	(25%)	(90 days)	(22.5, 2.69 IQR)	NR
					•	ICM with previous MI or known CAD (40%)					
					•	Congenital/inherited heart disease (13%)					
Bhaskaran et al.^11^	8	R	Single-center, Australia	Westmead Hospital (November 2013–NR)	•	Explanted ICD due to infection (38%)	NR	NR	36% ± 18% (28%)	100 ± 78 days (77 days)	23.4 ± 0.6 (23.3, 23.0–23.7)	NR
					•	DCM (25%)					
					•	Aortic stenosis (13%)					
					•	Myocarditis (13%)					
					•	Post-partum cardiomyopathy (13%)					
Kondo et al.^12,¶^	24	R	Single-center, Germany	University Hospital of Bonn (August 2010–November 2014)	•	Early post-MI for primary prevention (46%)	69 ± 12 years	92%	(30%)	(33 days)	(23.1, 21.6–23.6)	NR
					•	History of VT/VF (54%)					
Sasaki et al.^13^	9	R	Single-center, Japan	Hirosaki University Hospital (April 2014–September 2014)	•	History of VT/VF (89%)	(56 years)	78%	(35%)	(21 days)	(23.7, 23.6–23.9)	NR
					•	Early post-MI with reduced EF (11%)					
Epstein et al.^14^	8,453	R	Multicenter, US	Zoll database (September 2005–July 2011)	•	Early post-MI (100%)^#^	63 ± 13 years	73%	NR	69 ± 61 days (57 days)	(21.8, NR)	NR
Saltzberg et al.^15,¶,||^ (a)	107	R	Multicenter, US	Zoll database (2003–June 2009)	•	Pregnancy-related cardiomyopathy (100%)^#^	31 ± 6 years	0%	22% ± 9%	124 ± 123 days (87 days)	18.3 ± 5.3 (20.4, NR)	14%
Saltzberg et al.^15,¶,||^ (b)	159	R	Multicenter, US	Zoll database (2003–June 2009)	•	Nonischemic or idiopathic DCM^**^ with no mention of pregnancy-induced cause (100%)	33 ± 7 years	0%	21% ± 8%	96 ± 83 days (76 days)	17.0 ± 6.2 (19.3, NR)	8%
Rao et al.^16,||^ (a)	119	R	Multicenter, US	Zoll database (2005–2010)	•	Inherited arrhythmias (100%)	38 ± 13 years	45%	57% ± 10%	(29 days)	(19, 10–22)	9%
Rao et al.^16,||^ (b)	43	R	Multicenter, US	Zoll database (2005–2010)	•	Congenital structural heart disease (100%)	37 ± 12 years	47%	37% ± 19%	(27 days)	(19, 12–21)	9%
Chung et al.^17,¶¶^	2,274	R	Multicenter, US	Zoll database (August 2002–December 2006)	•	Explanted ICD (28%)	59 ± 15 years	74%	NR	52.6 ± 69.9 days (36 days)	19.9 ± 4.7 (21.7, NR)	14%
					•	Recent NICM with EF ≤ 35% (24%)					
					•	History of VT/VF (19%)					
					•	Post-CABG with EF ≤ 35% (11%)					
					•	Unspecified cardiomyopathy with EF ≤ 35% (10%)					
					•	Miscellaneous or unknown (8%)					
Klein et al.^18^	354	R	Single-center, Germany	University Hospital in Magdeburg (January 2000–June 2008)	•	Complicated^§§^ early post-MI (39%)	58 ± NR years	82%	NR	106 ± NR days	21.3 ± NR	4%
					•	Complicated^***^ post-CABG (25%)					
					•	Myocarditis/DCM (10%)					
					•	Explanted ICD due to infection (10%)					
					•	Heart transplant candidate (6%)					
					•	Syncope of unknown origin/aborted SCA (5%)					
					•	Long QT/Brugada syndrome (3%)					
					•	Delay/refusal of ICD implantation (2%)					
Feldman et al.^19^	289	P	Multicenter, US, and single-center, Germany	WEARIT and BIROAD studies (NR)	•	NYHA class III/IV HF with EF < 30% (61%, WEARIT population)	55 ± 12 years	82%	23% ± 10%	93 ± NR days	NR	24%
					•	Complicated^†††^ early post-MI or post-CABG; ICD candidate not expected to receive the device for at least four months; or ICD implantation refusal (39%, BIROAD population)					

BIROAD: Bridge to ICD in Patients at Risk of Arrhythmic Death; CABG: coronary artery bypass graft; CAD: coronary artery disease; DCM: dilated cardiomyopathy; EF: ejection fraction; ICD: implantable cardioverter-defibrillator; ICM: ischemic cardiomyopathy; MI: myocardial infarction; NICM: nonischemic cardiomyopathy; NR: not reported; P: prospective; R: retrospective; SCA: sudden cardiac arrest; SD: standard deviation; US: United States; VF: ventricular fibrillation; VT: ventricular tachycardia; WCD: wearable cardioverter-defibrillator; WEARIT: Wearable Defibrillator Investigative Trial; WIF: Wearable Defibrillator Use in Heart Failure; NR: not reported.

^*^Due to comfort issues, nonadherence, adverse reactions.

^†^German national database maintained by Zoll; all other Zoll databases in table used US data.

^‡^Including patients post-MI within 40 days and post-revascularization within 90 days.

^||^Patients included if early post-MI with an EF ≤ 35% or had a diagnosis code for acute MI (ICD-9 code: 410.xx).

^#^Results only reported for specific cohorts separately.

^**^Patients included if they had a diagnosis code for cardiomyopathy (ICD-9 codes: 425.4, 425.8); peri-partum cardiomyopathy (ICD-9 codes: 674.5, 674.45, 674.8); or secondary cardiomyopathy (ICD-9 code: 425.9) coupled with a reference to current or recent pregnancy (six or less months since last pregnancy).

^¶^Results only reported for specific cohorts separately.

^¶¶^Patients included if they had a diagnosis code for cardiomyopathy (ICD-9 codes: 425.4, 425.8) or secondary cardiomyopathy (ICD-9 code: 425.9).

^§§^Baseline demographics from entire study population.

^***^Pre- or early postoperative poor ventricular function, prolonged and difficult postoperative hemodynamic recovery, or early post-operative life-threatening ventricular tachyarrhythmias.

^†††^Ventricular tachyarrhythmia within 48 hours of the event, EF < 30% at least three days after the event, or an episode of syncope or sudden cardiac arrest at least 48 hours after the event but without candidacy for an ICD.

**Table 3:** Endpoint Results of Included Studies

Study^*^	Number of Participants (Number of Person-years)	All-cause Mortality^†^	VT/VF-related Mortality^†^	VT/VF Occurrence		Appropriate Shock Therapy		Successful Termination	Inappropriate Shock Therapy

		Deaths/Total N Incidence, % (95% CI)	Number of Deaths/Total Patients, Incidence (95% CI)	Number of Patients Affected/Total Patients, Incidence (95% CI)	Number of Patients Affected/100 PY, Rate (95% CI)	Number of Patients Affected/Total Patients, Incidence (95% CI)	Number of Patients Affected/100 PY, Rate (95% CI)	Number of Patients Affected/Number of Patients with Appropriate Shocks, Incidence (95% CI)	Number of Patients Affected/Total Patients, Incidence (95% CI)
Wäßnig et al.^5^	6,043 (1,124)	NR	7 deaths, 0.12% (0.05%–0.24%)	164 patients, 2.71% (2.32%–3.16%)	NR	94 patients, 1.56% (1.26%–1.90%)	94 patients, 8.40 (6.80–10.20)	88 patients, 93.62% (86.62%–97.62%)	26 patients, 0.43% (0.28%–0.63%)
Kutyifa et al.^6^	2,000 (544)	3, 0.15% (0.03%–0.44%)	0 deaths, 0.00% (0.00%–0.18%)	41 patients, 2.05% (1.48%–2.77%)	120 patients, 22.00 (18.24–26.31)	22 patients, 1.10% (0.69%–1.66%)	22 patients, 4.04 (2.53–6.12)	22 patients, 100% (84.56%–100%)	10 patients, 0.50% (0.24%–0.92%)
Bhaskaran et al.^11^	8 (2)	0, 0.00% (0.00%–36.94%)	0 deaths, 0.00% (0.00%–36.94%)	NR	NR	0 patients, 0.00% (0.00%–36.94%)	0 patients, 0.00 (0.00–168.44)	N/A	0 patients, 0.00% (0.00%–36.94%)
Kondo et al.^12,‡^	24 (2)	0, 0.00% (0.00%–14.25%)	OE	OE	3 patients, 138.25 (28.51–404.02)	OE	OE	OE	OE
Sasaki et al.^13^	9 (0.5)	1, 11.11% (0.28%–48.25%)	0 deaths, 0.00% (0.00%–33.63%)	1 patient, 11.11% (0.28%–48.25%)	1 patient, 192.31 (4.87–1,071.47)	1 patient, 11.11% (0.28–48.25)	1 patient, 192.31 (4.87–1,071.47)	1 patient, 100% (2.50%–100.00%)	0 patients, 0.00% (0.00%–33.63%)
Epstein et al.^14^	8,453 (1,320)	93, 1.10% (0.89%–1.35%)	18 deaths, 0.21% (0.13%–0.34%)	136 patients, 1.61% (1.35%–1.90%)	149 patients, 11.29 (9.55–13.25)	133 patients, 1.57% (1.32%–1.86%)	133 patients, 10.08 (8.44–11.94)	NR	99 patients, 1.17% (0.95%–1.42%)
Saltzberg et al.^15,‡,¶^ (a)	107 (25)	OE	OE	OE	OE	OE	OE	OE	0 patients, 0.00% (0.00%–3.39%)
Saltzberg et al.^15,‡,¶^ (b)	159 (33)	OE	OE	OE	OE	OE	OE	OE	0 patients, 0.00% (0.00%–2.29%)
Rao et al.^16,¶^ (a)	119 (11)	2, 1.68% (0.20%–5.94%)	0 deaths, 0.00% (0.00%–3.05%)	2 patients, 1.68% (0.20%–5.94%)	3 patients, 27.00 (5.57–78.91)	2 patients, 1.68% (0.20%–5.94%)	2 patients, 18.00 (2.18–65.03)	2 patients, 100% (15.8%–100%)	4 patients, 3.36% (0.92%–8.38%)
Rao et al.^16,¶^ (b)	43 (3)	2, 4.65% (0.57%–15.81%)	0 deaths, 0.00% (0.00%–8.22%)	0 patients, 0.00% (0.00%–8.22%)	0 patients, 0.00 (0.00–116.00)	0 patients, 0.00% (0.00%–8.22%)	0 patients, 0.00 (0.00–116.00)	N/A	0 patients, 0.00% (0.00%–8.22%)
Chung et al.^17,§^	2,274 (328)	19, 0.84% (0.50%–1.30%)	6 deaths, 0.26% (0.10%–0.57%)	49 patients, 2.15% (1.60%–2.84%)	68 patients, 20.75 (16.11–26.31)	49 patients, 2.15% (1.60%–2.84%)	49 patients, 14.95 (11.06–19.77)	48 patients, 97.96% (89.15%–99.95%)	NR
Klein et al.^18^	354 (103)	NR	NR	27 patients, 7.63% (5.09%–10.90%)	228 patients, 221.77 (193.91–252.50)	11 patients, 3.11% (1.56%–5.49%)	11 patients, 10.70 (5.34–19.14)	11 patients, 100% (71.51%–100%)	NR
Feldman et al.^19^	289 (75)	12, 4.15% (2.16%–7.14%)	NR	NR	NR	6 patients, 2.08% (0.77%–4.46%)	6 patients, 7.99 (2.93–17.39)	NR	6 patients, 2.08% (0.77–4.46)
**I^2^ (95% CI)**		**84% (70%–90%)**	**41% (0%–72%)**	**86% (74%–91%)**	**97 (96–98)**	**43% (0%–71%)**	**75 (46–85)**	**0% (0%–61%)**	**78% (54%–86%)**
**Pooled Effect (95% CI)**		**1.40 (0.68–2.38)**	**0.15 (0.07–0.26)**	**2.59 (1.83–3.49)**	**47.20 (29.81–64.60)**	**1.70 (1.39–2.05)**	**9.06 (6.22–11.90)**	**95.49 (92.04–98.00)**	**0.86 (0.45–1.40)**
**Egger’s p-value**		**0.35**	**0.77**	**0.32**	**0.16**	**0.46**	**0.52**	**0.50**	**0.80**

CI: confidence interval; N/A: not applicable; NR: not reported; OE: overlapping endpoint (data not utilized due to overlap with another included study); PY: person-years; VF: ventricular fibrillation; VT: ventricular tachycardia.

^*^Estimated based on sample size and median/mean follow-up when PYs not reported.

^†^During time of prescribed use of the WCD.

^‡^Study included for select nonoverlapping endpoints only.

^¶^Results only reported for specific cohorts separately.

^§^Data on patients post-MI and those with genetic disease were excluded due to overlap with other included studies.
